# High Fidelity Deep Sequencing Reveals No Effect of ATM, ATR, and DNA-PK Cellular DNA Damage Response Pathways on Adenovirus Mutation Rate

**DOI:** 10.3390/v11100938

**Published:** 2019-10-11

**Authors:** Jennifer Risso-Ballester, Rafael Sanjuán

**Affiliations:** Institute for Integrative Systems Biology (I2SysBio), Universitat de València-CSIC, Paterna, 46980 València, Spain; jennyrisso@mail.ucv.es

**Keywords:** mutation rate, DNA damage response, experimental evolution, Human Adenovirus Type 5

## Abstract

Most DNA viruses exhibit relatively low rates of spontaneous mutation. However, the molecular mechanisms underlying DNA virus genetic stability remain unclear. In principle, mutation rates should not depend solely on polymerase fidelity, but also on factors such as DNA damage and repair efficiency. Most eukaryotic DNA viruses interact with the cellular DNA damage response (DDR), but the role of DDR pathways in preventing mutations in the virus has not been tested empirically. To address this goal, we serially transferred human adenovirus type 5 in cells in which the telangiectasia-mutated PI3K-related protein kinase (ATM), the ATM/Rad3-related (ATR) kinase, and the DNA-dependent protein kinase (DNA-PK) were chemically inactivated, as well as in control cells displaying normal DDR pathway functioning. High-fidelity deep sequencing of these viral populations revealed mutation frequencies in the order of one-millionth, with no detectable effect of the inactivation of DDR mediators ATM, ATR, and DNA-PK on adenovirus sequence variability. This suggests that these DDR pathways do not play a major role in determining adenovirus genetic diversity.

## 1. Introduction

In viruses, the rate at which new mutations are produced varies amply from 10^−8^ to 10^−4^ substitutions per nucleotide per cell infection cycle (s/n/c) [1]. Within this range, the highest mutation rates (10^−6^–10^−4^ s/n/c) correspond to RNA viruses, whereas DNA viruses mutate more slowly (10^−8^–10^−6^ s/n/c) [2]. As a result, RNA virus populations are highly diverse and evolve rapidly in response to a variety of selective pressures including immunity and antiviral treatments [3,4], whereas DNA virus populations tend to be less diverse, with some exceptions [5,6]. Therefore, knowledge of the mechanisms underlying viral mutation rates results in a better understanding of the ability of viruses to evolve, infect new hosts, and evade host cell responses.

It is widely accepted that the absence of a 3′exonuclease proofreading activity in most RNA-dependent RNA polymerases (RdRp) is a major factor responsible for RNA virus high mutability [2]. RdRp are unable to correct mistakes during replication, unlike many DNA polymerases that replicate DNA virus genomes. However, viral mutation rates are not only affected by replication, but also by other factors such as nucleic acid editing and spontaneous damage. Accordingly, access to cellular post-replicative repair is another factor that might explain the differences in mutation rate observed between DNA and RNA viruses. Cellular post-replicative repair cannot be used by RNA viruses but might be used by DNA viruses. This was investigated, for instance, in *Escherichia coli* and bacteriophage ϕX174. A major post-replicative system in *E. coli* is methyl-directed mismatch repair, which utilizes methylation of GATC sequence motifs to identify the parental strand, a step required to excise incorrect bases from the daughter strand [7]. The ϕX174 genome has evolved to contain no GATC motifs, preventing this type of repair and increasing the viral mutation rate [8]. Analogously, the mutation rate of eukaryotic DNA viruses could in principle be determined by interactions with the cellular DDR, yet despite virus-DDR interactions being amply reported [9,10], whether DDR determines the viral mutation rate remains unclear.

The DDR consists of a regulatory network of cellular pathways that detect and repair DNA damage in order to maintain genome integrity. Three key proteins of the DDR are the ataxia-telangiectasia-mutated PI3K-related protein kinase (ATM), the ATM/Rad3-related (ATR) kinase, and the DNA-dependent protein kinase (DNA-PK) [11,12,13]. ATM and DNA-PK control double-strand-break repair through homologous recombination and non-homologous end-joining (NHEJ), while ATR mainly responds to single-stranded DNA exposure [14,15,16]. A complex interplay between DDR and some DNA viruses has been demonstrated, in which viruses repress or activate specifically DDR components to promote viral replication [17,18,19,20]. For instance, herpes simplex virus [21,22,23], polyomaviruses [24], and simian virus 40 [25,26] activate and exploit DDR pathways, and their replication is thus less efficient in DDR-negative cells. In contrast, other viruses suppress DDR through different strategies, including mislocalization and degradation of DDR components [27].

Human adenoviruses exhibit complex interactions with DDR signaling. For instance, the E4orf3 protein mislocalizes the MRN complex, which is involved in the initial processing of double-strand DNA breaks, leading to ATM and ATR signaling inhibition [28,29]. In turn, expression of the adenovirus E4orf6/E1b55K complex promotes degradation of Mre11, a component of the MRN complex, as well as other enzymes required for DNA repair [30,31,32]. In previous work, we inferred a mutation rate of 1.3 × 10^−7^ s/n/c for human adenovirus 5 (Ad5) in HeLa cells [33]. This value was within the order of magnitude of mutation rates typically exhibited by tumor cells [34] but, because tumor cells are aberrant for DNA repair, it was difficult to ascertain whether the DDR shutdown plays a role in determining Ad5 mutation.

Here, we have used high-fidelity deep sequencing to test the effect of ATM, ATR, and DNA-PK shutdown on the production of viral genetic diversity via new mutations in Ad5. For this, we serially transferred Ad5 in DDR-proficient human lung fibroblasts (MRC-5) as well as in MRC-5 cells chemically inhibited for the three main DDR kinases. We found no effect of ATM, ATR, and DNA-PK kinase impairment on the genetic diversity accumulated in these experimental populations, suggesting that these DDR pathways play no major role in determining adenovirus mutation rates.

## 2. Materials and Methods 

### 2.1. Virus and Cells

MRC-5 primary human lung fibroblasts and HeLa-H1 cells (CRL-1958) were obtained from the American Type Culture Collection (references CCL-171 and CRL-1958, respectively). Cells were cultured at 37 °C and 5% CO_2_ in Dulbecco modified Eagle medium (DMEM) supplemented with 10% fetal bovine serum (FBS) and antibiotics (penicillin-streptomycin). Mycoplasma contamination was routinely ruled out by PCR. Ad5 was a generous gift from Dr. Ramón Alemany (Bellvitge Biomedical Research Institute).

### 2.2. DDR Kinase Inhibition

ATM inhibitor KU55933 (Sigma-Aldrich, Misuri, USA), ATR inhibitor VE-821 (ApexBio, Houston, USA), and DNA-PK inhibitor NU7441 (ApexBio) were dissolved in DMSO to obtain concentrated stocks. DDR inhibition was carried out by pretreating cells with the indicated final concentration of each inhibitor for 2 h and keeping the inhibitor present during the entire infection assay. To induce DNA damage, cells were treated with neocarzinostatin (NCS; 0.5 mg/mL, Sigma-Aldrich) for 30 min.

### 2.3. Cytotoxicity

Cells were treated with the indicated DDR kinase inhibitor, a blank without cells was performed and as a negative control, DMSO was added to cells. After 72 h of incubation, resazurin (Sigma-Aldrich) was added to a final concentration of 0.1 mM and cells were incubated for 4 h in total darkness. Fluorescence was recorded between 540 nm–590 nm using a SPARK plate reader (TECAN). These assays were performed in triplicate in 96-well plates.

### 2.4. DDR Activation Measured by Immunofluorescence

Cells were seeded in 6-well plates or 12 mm circular coverslips in 12-well plates. After treatment with the corresponding compound, fresh media was aspirated and cell monolayers were washed with PBS for 5 min three times. Cells were fixed with 4% paraformaldehyde for 15 min at 4 °C and permeabilized with 0.1% Triton X-100 in PBS for 10 min at room temperature. Cells were rinsed with PBS three times, blocked with blocking solution (1% BSA, 10% goat serum in 0.1% PBS-Tween) for 1 h and stained with the primary antibody in blocking solution for 16 h at 4 °C. After PBS washing, cell monolayers were stained with the secondary antibody diluted in blocking buffer for 1 h at room temperature in darkness. Cell nuclei were stained with DAPI (Sigma-Aldrich) at a final concentration of 0.1 µg/mL for 15 min at RT. The primary antibodies targeted H2AX histone phosphorylation at Ser 139 (γ-H2AX; 1:200; Merck Millipore, Massachusetts, USA), ATM phosphorylation at Ser 1981 (1:200; Santa Cruz Biotechnology, Dallas, USA), DNA-PK phosphorylation at Ser 2056 (1:200; Abcam, Cambridge, UK), and Chk-1 phosphorylation at Ser 345 (1:50; Cell Signaling, Danvers, USA). The secondary antibodies were Alexa488 goat anti-mouse IgG (1:1000; Abcam) and Alexa488 goat anti-rabbit IgG (1:1000; Abcam). Fluorescence was quantified by high-content screening (INCell 2200, General Electrics, New York, USA).

### 2.5. Virus Titration

The virus was titrated by the plaque assay in HeLa H1 cells. Plaque assays were not conducted in MRC-5 cells because the virus did not produce visible plaques in this cell line under our assay conditions. A virus inoculum (100 µL in 6-well plates) was added to 70–80% confluent cells and after 4 h at 37 °C, cells were washed with PBS and incubated for 5 days at 37 °C with 5% CO2 in a semi-solidified medium containing DMEM supplemented with 1% FBS, 1% penicillin-streptomycin, and 0.8% noble agar and a nutrient medium layer of DMEM supplemented with 1% FBS and 1% penicillin-streptomycin. After incubation, cell monolayers were fixed with 10% formaldehyde and stained with 2% crystal violet. Viral titers were expressed as plaque-forming (PFU) per mL.

### 2.6. Serial Virus Transfers

Ad5 was passaged in MRC-5 cells at a multiplicity of infection (MOI) of 0.1 PFU/cell under the indicated conditions. In each passage, the viral population was allowed to grow for 72 h. After incubation, cell monolayers and supernatants were collected and cells were lysed by three consecutive freeze-thaw steps. Virus samples were titrated by the plaque assay in HeLa H1 cells and conveniently diluted to infect fresh cells until passage 10. For each condition, three independent evolution lines were performed. 

### 2.7. Viral DNA Purification

Cells were detached using trypsin, harvested by centrifugation, washed with PBS, resuspended in DMEM, and lysed by three free-thaw cycles. Then, cellular debris was removed by centrifugation and the supernatant was treated with DNAse I for 30 min at 37 °C. Following DNAse I heat inactivation, samples were treated with RNAse A for 1 h at 37 °C. Subsequently, viral lysis buffer (10% SDS, 0.5 M EDTA, and 10 mg/mL proteinase K) was added for disrupting viral capsids for 1 h at 56 °C. Viral DNA was extracted with phenol/chloroform, resuspended in ddH2O, quantified using the Qubit dsDNA HS Assay Kit (Life Technologies, California, USA), and used directly for Illumina duplex sequencing. A pUC18 plasmid DNA purified with the NucleoSpin Plasmid kit (Macherey-Nagel, Duren, Germany) was used as sequencing control.

### 2.8. Duplex Sequencing

The purified viral DNA (500 ng) was fragmented by sonication (Covaris S2 Sonication System) and fragments were selected by size with Ampure X beads, used for ligating duplex sequencing adaptors, and run on a NextSeq machine (Illumina, California, USA) with a read length of 2 × 150 bp. The duplex sequencing adaptors contain random double-stranded nucleotide sequences, which allow tracing each strand of the original double-stranded template. Sequencing errors are removed by constructing a consensus sequence for each group of reads sharing the same adaptor sequence (a “family” originated from the same template). A consensus is built for each strand, and finally, duplex consensus sequences (DCS) are obtained by combining consensus sequences with complementary adaptors. For this, FASTQ files were processed using the duplex sequencing pipeline (https://github.com/loeblab/Duplex-Sequencing), BWA 0.7.17, Samtools 1.5, Picardtools 2.20.2 and GATK 4.0. The UnifiedConsensusMaker.py DS script was used to transform BAM files into two FASTQ files with their DCS. The DCS were aligned to the reference genome AY601635 to analyze each genome position and finally, count mutations. Default values were used for all parameters except for the family size required to build consensus reads, which was set to 2. Increasing the family size to 3 yielded similar results. Specifically, we obtained slightly lower coverage and fewer polymorphic nucleotide sites (50–188), the average mutation frequencies being (5.5 ± 0.7) × 10^−6^, (6.6 ± 0.1) × 10^−6^ and (6.2 ± 1.6) × 10^−6^ s/n for the founder virus, the virus evolved in untreated cells, and the virus evolved in DDR-inhibited cells, respectively. Details of the duplex sequencing protocol and pipeline have been described previously [35]. The DS output of each sample is available from the NCBI SRA database (www.ncbi.nlm.nih.gov/sra), accession PRJNA561356.

### 2.9. Detection of Deletions

Two specific analyses were implemented to study this type of mutation. The first method has been described in previous work and is based on analyzing the mapping distance between paired-end reads [36]. In our libraries, the expected size of Illumina inserts was 300 bp and the size of paired-end reads was 150 bp. We mapped all paired-end reads to the reference genome using Bowtie 2 (http://bowtie-bio.sourceforge.net/index.shtml) and obtained the distance between each pair of reads. Distances larger than the expected value were indicative of a deletion. The second method was based on directly identifying discontinuities in the mapping positions of each read, without using paired-end information. All reads were mapped to the reference sequence using Bowtie 2, mapped reads were extracted from BAM files and the mapping position of each read base was obtained. Large gaps in the list of mapping positions suggested a deletion. To avoid mapping errors due to poor read quality, the minimal Phred quality score was set to 30. For both approximations, reads were mapped to the reference genome AY601635.

## 3. Results

### 3.1. DDR Pathway Inhibition

The three main DDR kinases were inactivated to study a possible relationship between these DNA repair pathways and the accumulation of adenovirus genetic diversity. For this, we used KU55933, a compound that selectively inhibits ATM activation [37], VE-821, which inhibits ATR activation [38], and NU7441, which inhibits DNA-PK activation [39]. The cytotoxicity of each inhibitor was tested by subjecting MRC-5 cells to different drug concentrations for 72 h. We selected a combination of 10 µM KU55933, 1 µM NU7441, and 5 µM VE-821 to inhibit the three pathways without compromising cellular viability excessively (Figure S1). 

To verify the efficacy of these chemical inhibitors, we stimulated DNA strand break with different concentrations (0–1000 ng/mL) of neocarzinostatin (NCS) for 30 min [40,41]. This antitumor drug induces single and double-stranded DNA breaks (SSBs and DSBs) by free radical mechanisms [40,41]. H2AX histone phosphorylation at Ser 139 (γ-H2AX) was used as a DNA damage marker [42,43]. ATM kinase is considered the main mediator of H2AX phosphorylation in response to DSBs [44,45]. However, H2AX can also be phosphorylated by ATR in response to SSBs or during replication stress [46,47] and by DNA-PK [48,49]. In addition, DNA damage caused by ionizing radiation leads to H2AX phosphorylation by the three PIKK kinases ATM, ATR, and DNA-PK [50].

A direct correlation between NCS dosage and γ-H2AX fluorescence signal was obtained (Figure S2). Treatment of cells with 1000 ng/mL for 30 min resulted in strong DNA damage induction and was used to subsequently test the efficiency of each DNA repair kinase inhibitor.

**Figure 1:**
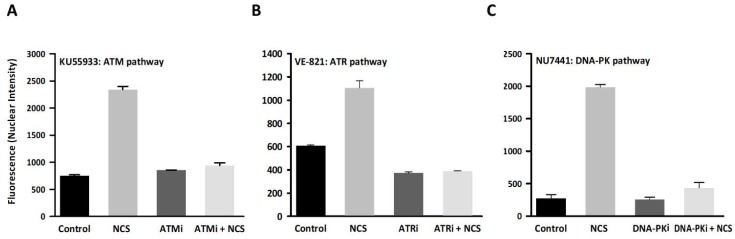
The ability of chemical inhibitors to suppress activation of DNA damage response (DDR) kinases ATM, ATR, and DNA-PK. MRC-5 cells were untreated (Control), treated with the DNA damage inductor NCS, treated with the indicated DDR kinase inhibitor only (ATMi, ATRi or DNA-PKi), or treated with both (neocarzinostatin (NCS) + DDR kinase inhibitor). DDR activation was assayed by fluorescence-based immunocytochemistry using high-content screening to measure fluorescence intensity. The DDR kinase inhibitors used were KU55933 for ATM (**A**), VE-821 for ATR (**B**), and NU7441 for DNA-PK (**C**). Activation of their corresponding targets was determined using antibodies against ATM phosphorylated at Ser1981 (**A**), Chk1 phosphorylated at Ser345 (**B**), or DNA-PK phosphorylated at Ser2056 (**C**). Data are shown as the mean of two independent assays. Fluorescence intensity was measured in 100–300 nuclei foci for each experimental condition. Error bars represent the standard error of the mean.

Following treatment with each kinase inhibitor (KU55933, VE-821, or NU7441), DNA damage was induced with NCS and the activation of each DNA repair protein (ATM, ATR, and DNA-PK, respectively) was quantified by immunofluorescence (Figure 1; Figure S5). NCS induced the three DDR pathways, ATM, ATR, and DNA-PK (*t*-tests: *p* < 0.001 in all cases). Moreover, we found that DDR pathways were suppressed efficiently by each of the three assayed compounds, since activation of ATM, ATR, and DNA-PK was significantly reduced (*t*-tests: *p* < 0.001 in all cases) and nearly recovered basal values.

### 3.2. Effect of DDR Inhibition on the Accumulation of Genetic Diversity in Ad5

We tested the effect of the DDR kinase inhibitors on the genetic diversity of experimental Ad5 populations by subjecting the virus to 10 serial transfers using an MOI of 0.1 PFU/cell at inoculation. To ensure that no pre-existing genetic diversity was present, before initiating the serial transfers, the virus was subjected to three consecutive limiting dilution steps to isolate a single infectious unit. Three independent evolution experiments were carried out, each started from a single infectious unit obtained by limiting dilution. The evolution was done in untreated MRC-5 cells or in cells treated with the above combination of ATM, ATR, and DNA-PK inhibitors (KU-55933 + VE-821 + NU7441). After transfer 10, Ad5 DNA was extracted and used directly (i.e., without PCR amplification) for high-fidelity high-throughput sequencing by the duplex sequencing method with Illumina technology. We also sequenced the three founder viruses. Hence, nine Ad5 populations were sequenced in total. In addition, we sequenced a standard bacterial plasmid (pUC18) as a control for sequencing accuracy.

We obtained an average coverage of 1191 reads per base, with 94.5% of the genome positions showing coverage values higher than 500, which allowed us to investigate the diversity of each viral population in depth using as a reference sequence the GenBank accession AY601635 (Figure S3). We found between 68 and 215 polymorphic nucleotide sites in the nine populations sequenced (Table 1, Table S1). Most mutations (68.2%) were observed in only one read and hence had population frequencies typically on the order 0.1%, whereas 31.8% of the mutations occurred multiple times and reached frequencies between 0.1 and 1%. Only 14 mutations were observed at frequencies >1%. Mutation frequency, calculated as the total mutation count divided by the total number of bases sequenced, ranged from 3.2 × 10^−6^ s/n to 8.3 × 10^−6^ s/n (Table 1). The mutation frequency of the evolved populations was slightly higher than that of the founders (*t*-test: *p* = 0.019), but there were no significant differences in mutation frequency between viruses evolved in DDR kinase-inhibited cells and untreated cells (*p* > 0.05). Mutation frequencies were low but nevertheless higher than the frequency observed in the pUC18 plasmid control (1.6 × 10^−6^; one-sample *t*-test: *p* < 0.001), indicating that most mutations were real and not mere sequencing artifacts.

**Figure 2:**
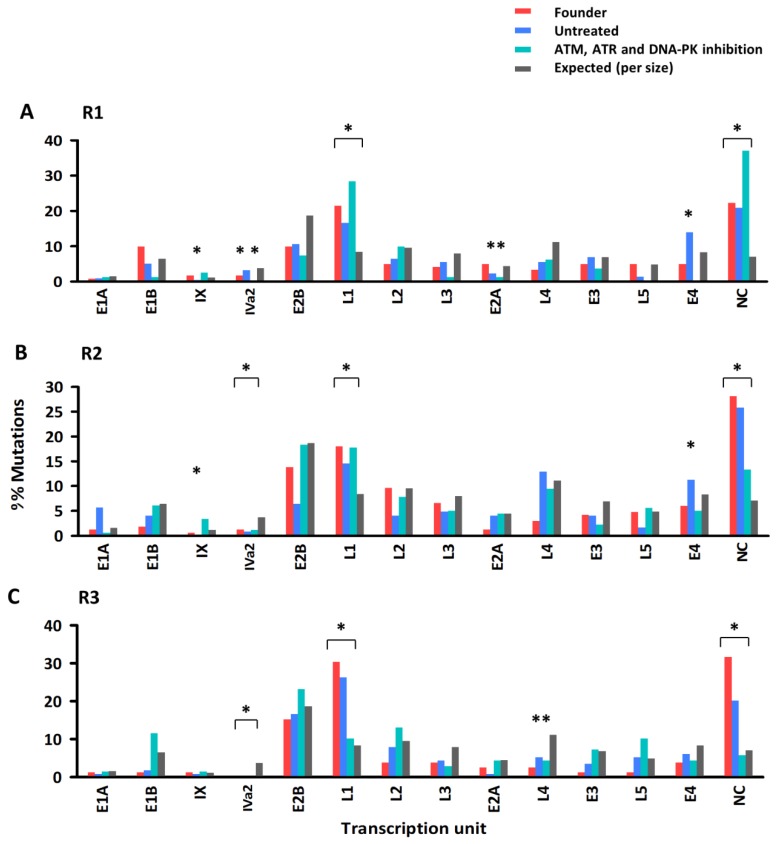
Mutation counts per adenovirus coding region. The number of mutations detected in each Ad5 transcriptional unit is shown for each evolution line (**A**: R1, **B**: R2, **C**: R3) and treatment, compared to the corresponding founder virus of this line. Observed mutation counts (colors) are shown versus expected counts assuming a constant genome mutation rate (grey). NC: non-coding regions (pooled). * *p* < 0.05 (Chi-square test).

The distribution of mutations along the genome was not fully uniform since in some cases, the observed number of mutations per transcription unit deviated from the number expected by chance, after taking the size of each region into account (Figure 2). Specifically, transcription unit L1 and non-coding regions showed an excess of diversity, whereas IVa2 showed less diversity than expected by sheer chance. Deviations from expected mutation counts were also observed for some populations in transcription units IX, L4, and E4. This suggests that mutations were not produced at a constant rate throughout the viral genome as shown previously [33], or that selection against deleterious mutations reduced observed mutation frequencies in some genes more than in others. However, the distribution of mutations along the viral genome was very similar for the founder viruses, lines evolved in untreated cells and lines evolved in DDR kinase-inhibited cells.

**Figure 3:**
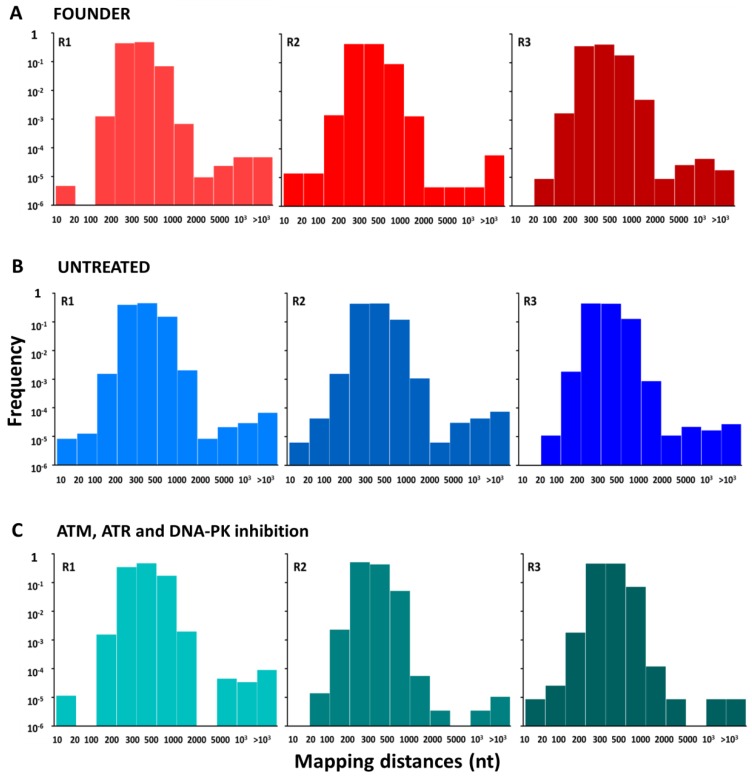
Distribution of deletion sizes for each of the nine sequenced adenovirus populations. The frequency of each gap size between paired-end reads is represented for each replicate of the founder virus (**A**) and each evolved virus population (untreated and DDR kinase triple inhibition) (**B**,**C**). The frequency of each gap size was calculated by dividing the count of gaps between an established size interval by the total number of gaps or paired-end reads analyzed.

Although small insertions and deletions were detected by the duplex sequencing method, their frequency was similar in viral sequences and in the control plasmid and hence, we cannot discard that these were sequencing artifacts. Furthermore, the duplex sequence pipeline is not designed for the detection of large deletions. To address this, we explored two alternative methods. First, we calculated the distance between paired-end reads. As shown previously [36], large distances between paired-end reads suggest the presence of deletions. Using this method, we did not find evidence for differences in deletion frequency or deletion size among treatments (Figure 3). Second, for each individual sequence read, we calculated the distance between the mapping positions of each consecutive site in the read. If no deletions were present, this distance should equal 1, whereas larger values suggest a deletion. Again, this method revealed no differences in the frequency or type of deletions among founder viruses, viruses evolved in untreated cells, and viruses evolved in cells treated with DDR kinase inhibitors (Figure S4). 

## 4. Discussion

Post-replicative repair and 3’exonuclease proofreading are two major mechanisms responsible for the genetic stability of the cell. Most DNA virus polymerases encode 3´exonuclease activity. Specifically, the domain organization of adenovirus polymerases has been studied previously, and the presence of 3´exonuclease activity has been demonstrated [51,52,53]. Furthermore, adenovirus mutator polymerases have been constructed by modifying the nucleotide-binding pocket or the exonuclease domain [54]. In contrast, whether DNA viruses can access post-replicative repair for correcting replication errors remains poorly investigated, particularly for eukaryotic viruses, including adenoviruses.

To address this question, we have used Ad5, a virus for which we previously estimated a mutation rate of 1.3 × 10^−7^ s/n/c in tumoral cells [33]. Using similar methods, here we obtained an average mutation frequency of (9.3 ± 0.7) × 10^−6^ s/n for three populations serially passaged 10 times in MRC-5 cells (without ATM, ATR, or DNA-PK inhibition). Mutation frequencies differ from mutation rates because they do not measure the number of new mutations produced per cell infection cycle but instead, the abundance of mutations in a given population at a given time point. Observed mutation frequencies depend on mutation rate, but also on the number of generations elapsed, selection, and other population genetic processes [55]. Counting unique mutations only (i.e., polymorphic nucleotides sites), the average mutation frequency for the viral population evolved in untreated cells was (3.4 ± 1.0) × 10^−6^ s/n. If we assume approximately two infectious cycles per transfer and ignoring selection, we obtain a mutation rate estimate of 3.4 × 10^−6^/10/2 = 1.7 × 10^−7^ s/n/c, a value similar to our previous estimate [33]. However, unexpectedly in the present study, mutation frequencies increased only modestly in evolved viruses compared to the founder viruses, indicating little accumulation of genetic diversity after 10 transfers. There are several possible explanations for this result. First, it is possible that the actual mutation rate of Ad5 in MRC-5 cells was extremely low, such that most of the observed changes were indeed sequencing artifacts. However, this possibility is contradicted by the lower mutation frequency observed in our control bacterial plasmid. Second, it is possible that most adenovirus mutations were highly deleterious or lethal and hence, failed to accumulate through transfers. If this was the case, one should not expect large differences between the observed mutation frequency and the actual mutation rate [1]. Yet, mutation frequencies were over 20-fold higher than our previously estimated mutation rate for this virus. Finally, it is possible that genetic diversity failed to accumulate because the virus experienced strong population bottlenecks between transfers, resulting in loss of genetic diversity under the action of random genetic drift. However, this seems unlikely given that an MOI of 0.1 PFU/cell was used, implying that each transfer was initiated with approximately 7 × 10^4^ infectious units.

We also failed to detect an effect of chemical inhibitors on observed Ad5 mutation frequencies despite the fact that these compounds efficiently blocked ATM, ATR, and DNA-PK DDR pathways. Different types of interactions between Ad5 and DDR components have been described previously [56], but our data provided no evidence that these interactions have an effect on Ad mutation rates. These results suggest that Ad5 mutations are DDR-independent, but alternative interpretations of our results are possible. For instance, it is possible that since Ad5 tends to block DDR pathways, our chemical treatments had little effect on DDR activity in infected cells beyond the virus-induced blockade. In future work, it would interesting to test the effects of over-activating DDR pathways on Ad5 mutation rate. Another possibility is that functional redundancy prevented us from efficiently suppressing interactions between the virus and the DDR system. Alternatively, chemically-induced inhibition of DDR pathways might have been incomplete and might have allowed for residual DDR kinases activity. This could be addressed in future work using RNAi or CRISRP gene knock-down techniques. In preliminary experiments though, we failed to obtain ATM negative MRC-5 cells by CRISPR. Finally, as discussed above, it is also possible that mutations were removed by genetic drift, or that excessive noise resulting from sequencing artifacts prevented us from detecting the effects of DDR components inhibition.

Yet another possibility is that our DDR inhibition promoted the appearance of new mutations, but that these mutations were highly deleterious and were rapidly counter-selected in the population, preventing their detection despite the high sequencing depth achieved. In future experiments, it might be informative to investigate the consequences of DDR inhibition for Ad5 infectivity, for instance, by determining the viral particle-to-PFU ratio. For this, the viral particle concentration has to be determined by physical methods and compared to the titer measured by the plaque assay. Particle concentrations can be quantified using transmission electron microscopy [57,58] or using more recent methods such as nanoparticle tracking analysis [59,60] and tunable resistive-pulse sensing [61]. An alternative approach to counting particles is to quantify viral DNA by qPCR, which provides an estimate of the genome-to-PFU [62].

In conclusion, our high-fidelity deep sequencing failed to reveal any effect of ATM, ATR, and DNA-PK inhibition on adenovirus mutation frequencies. This suggests that adenoviruses do not use these DDR pathways for correcting replication errors, although our results should be validated using alternative methods before conclusively discarding a role for these DDR pathways.

Insights into the consequences of the interaction between Ad and DDR on virus genetic diversity could be helpful for adenoviral vector development. Ad5 is the most common serotype used as a vector for gene therapy [63]. Ads are strongly immunogenic and this is often a problem for their use as vectors [64]. DDR pathways are also part of the host’s defense against viral infection. Viruses are recognized by the innate immune system, and if they contain DNA as genetic material, they can also cause DDR activation [27]. Hence, virus-DDR interactions might play a role in the genetic stability and efficacy of Ad vectors. Since some target cells might have DDR defects, it is important to assess whether changes in DDR activation might have an effect on vector genetic stability. Our results suggest that no major effects should expected.

## Figures and Tables

**Table 1:** Sequence diversity of Ad5 passaged in DDR-inhibited or untreated cells.

Experiment		1			2			3	
Virus population	Founder	Untreated	DDRi ^1^	Founder	Untreated	DDRi ^1^	Founder	Untreated	DDRi ^1^
Polymorphic sites	122	215	81	171	125	182	80	113	68
Total mutation count	159	396	118	272	243	320	96	408	92
Mbp sequenced	42.9	43.6	23.6	49.8	33.2	48.9	18.8	78.1	28.5
Mutation frequency (×10^6^)	2.8	4.9	3.4	3.4	3.8	3.7	4.3	1.4	2.4

^1^ Kinase DDR inhibition (ATM, ATR, and DNA-PK) using chemical inhibitors.

## Supplementary Materials

The following are available online at https://www.mdpi.com/1999-4915/11/10/938/s1, **Figure S1. Cytotoxicity of DDR kinase inhibitors.** Percent viability of MRC-5 cells treated with different concentrations of ATM inhibitor **(A)** ATR inhibitor **(B)** DNA-PK inhibitor **(C)** or the indicated combinations of the three inhibitors (**D**). Error bars represent the standard error of the mean from three independent replicates. The arrow indicates the concentration combination of inhibitors used in further assays; **Figure S2. Induction of DNA damage with NCS.** MRC-5 cells were treated with the indicated concentrations of NCS to induce DNA damage. Left: representative image showing γ-H2AX foci and their nuclear localization (co-localization with DAPI). Right: γ-H2AX nuclear fluorescence intensity in response to each NCS drug dosage determined by high-content screening. Each condition was assayed in triplicate. Fluorescence intensity was measured in 100–300 nuclei foci for each experimental condition. Error bars represent the standard error of the mean; **Figure S3. Sequencing coverage for each evolved line and the founder virus.** Each replicate of each experimental condition **(B and C)** and the founder virus **(A)** is shown. **Figure S4. Distribution of deletion sizes for each of the nine sequenced adenovirus populations.** Each replicate of the founder virus **(A)** and each experimental condition **(B and C)** is shown. The frequency of each map distance between consecutive sequencing reads is shown for each virus sample; **Figure S5. The ability of DDR kinase inhibitors to suppress ATM, ATR, and DNA-PK pathways in the presence of a genotoxic agent.** MRC-5 cells were treated with the appropriate DDR kinase inhibitor and then DNA damage was induced by exposing cells to 1000 ng/mL of NCS. After that, each DDR pathway activation was assayed by fluorescence-based immunocytochemistry using high-content screening to measure fluorescence intensity. Activation of their corresponding targets was determined using antibodies against ATM phosphorylated at Ser1981 (ATM pathway), Chk1 phosphorylated at Ser345 (ATR pathway), or DNA-PK phosphorylated at Ser2056 (DNA-PK pathway). Data are shown as the mean of two independent assays. Fluorescence intensity was measured in 100–300 nuclei foci for each experimental condition. Error bars represent the standard error of the mean; **Table S1. List of all mutations detected by duplex sequencing in each evolution line for a minimal consensus family size of two reads (Excel format).** This includes genome position in the reference sequence (AY601635), mutant base, sequence coverage, number of mutation-containing reads, strand and transcription unit, encoded protein (gene) and protein product, mutated codons, and amino acid substitution.
