# Supplementary figures and images for: High Fidelity Deep Sequencing Reveals No Effect of ATM, ATR, and DNA-PK Cellular DNA Damage Response Pathways on Adenovirus Mutation Rate
Source: Viruses. 2019 Oct 11;11(10):938. doi: 10.3390/v11100938 (PMC6832117; doi:10.3390/v11100938)

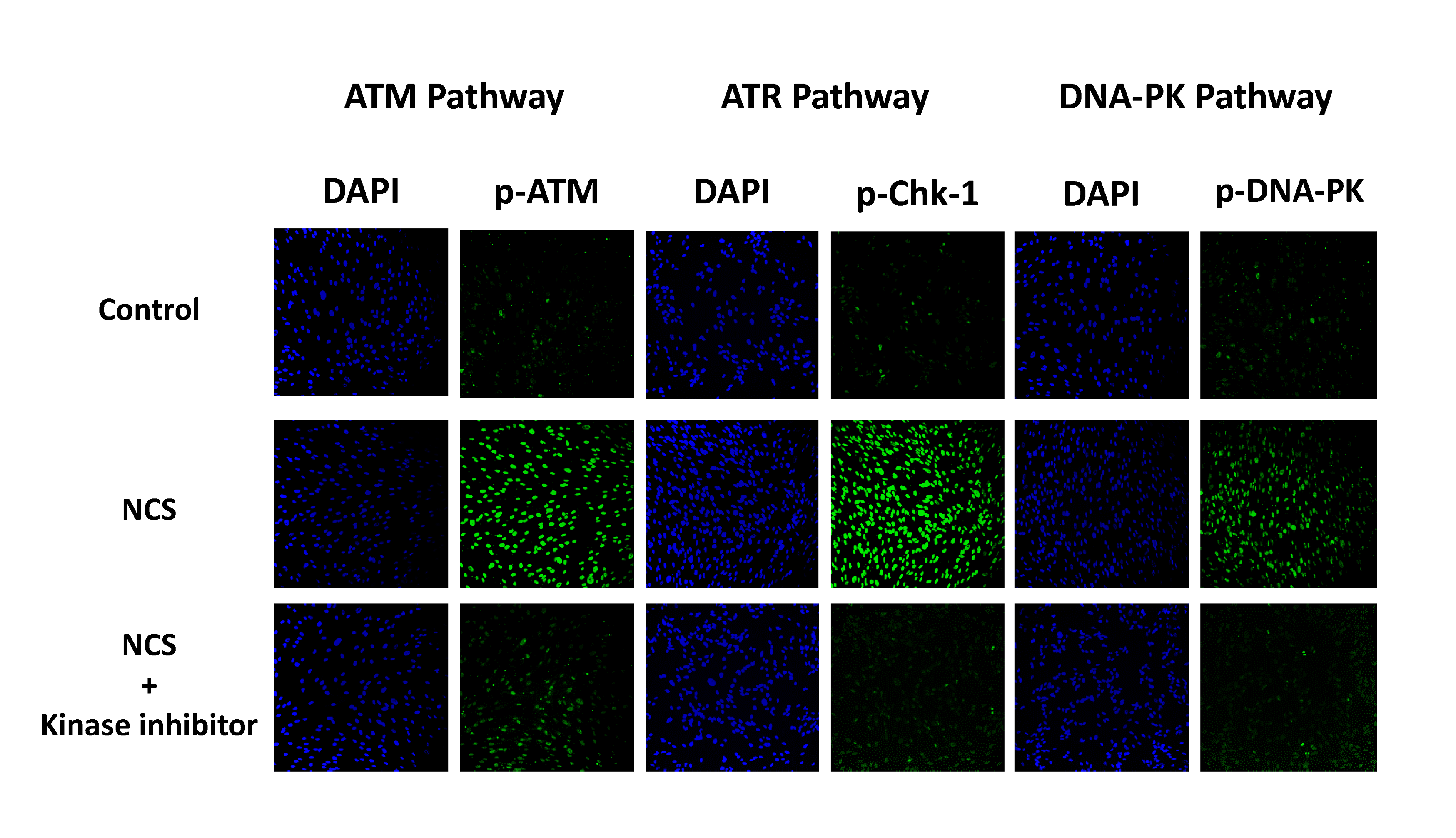

Supplement: Supplementary file 1 [file viruses-11-00938-s001.zip › Fig_S5.tiff]

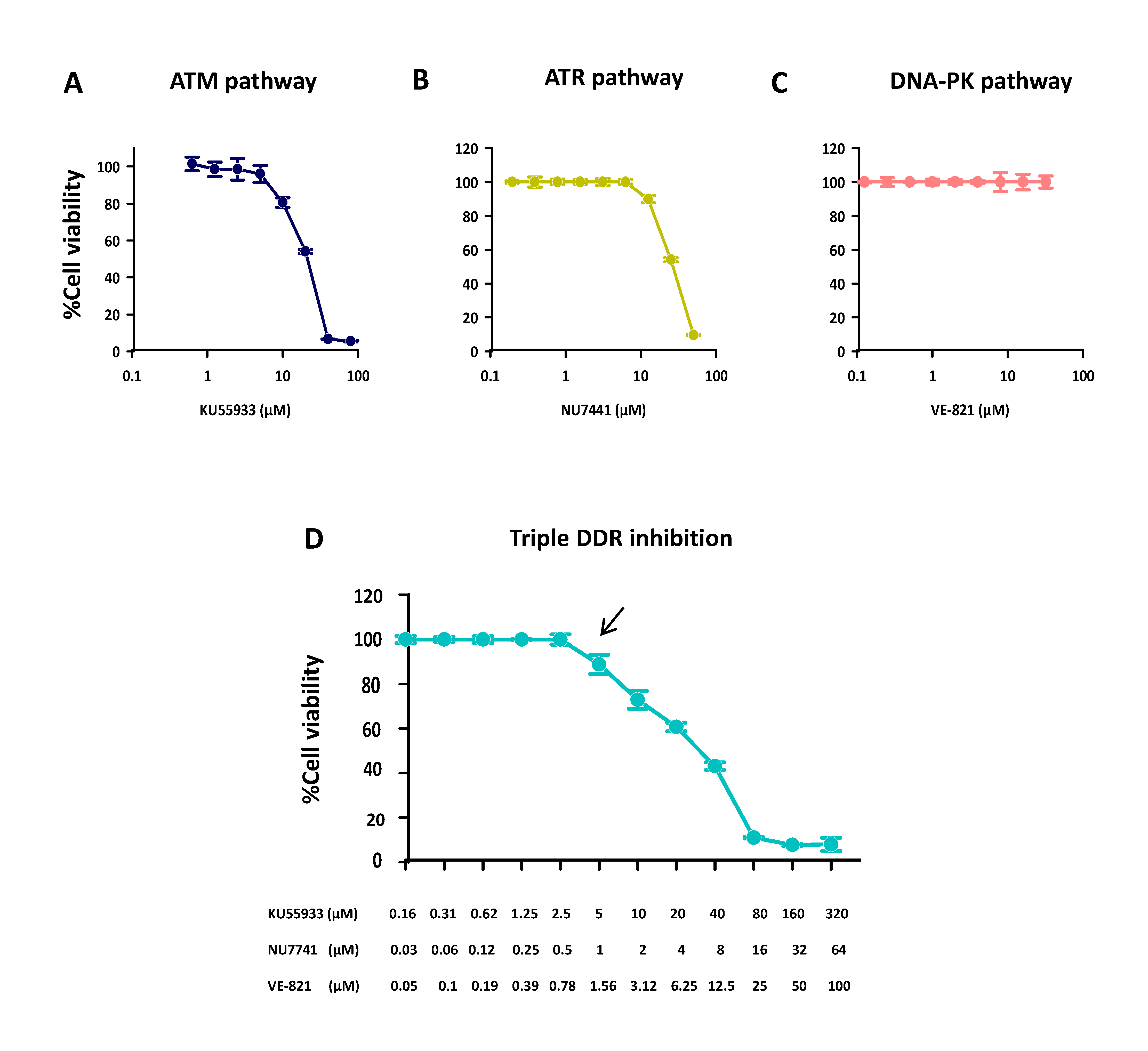

Supplement: Supplementary file 1 [file viruses-11-00938-s001.zip › Fig_S1.tiff]

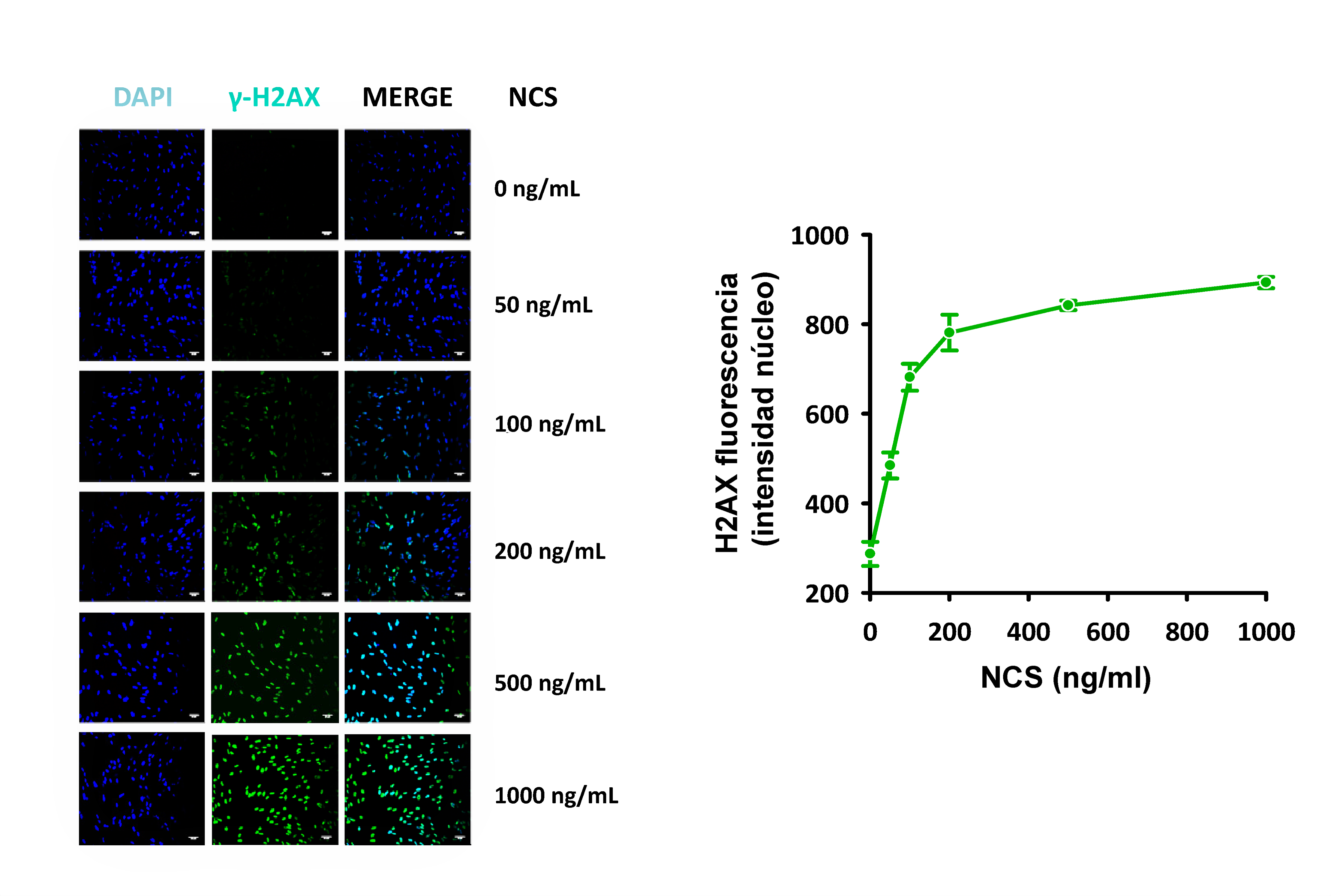

Supplement: Supplementary file 1 [file viruses-11-00938-s001.zip › Fig_S2.tiff]

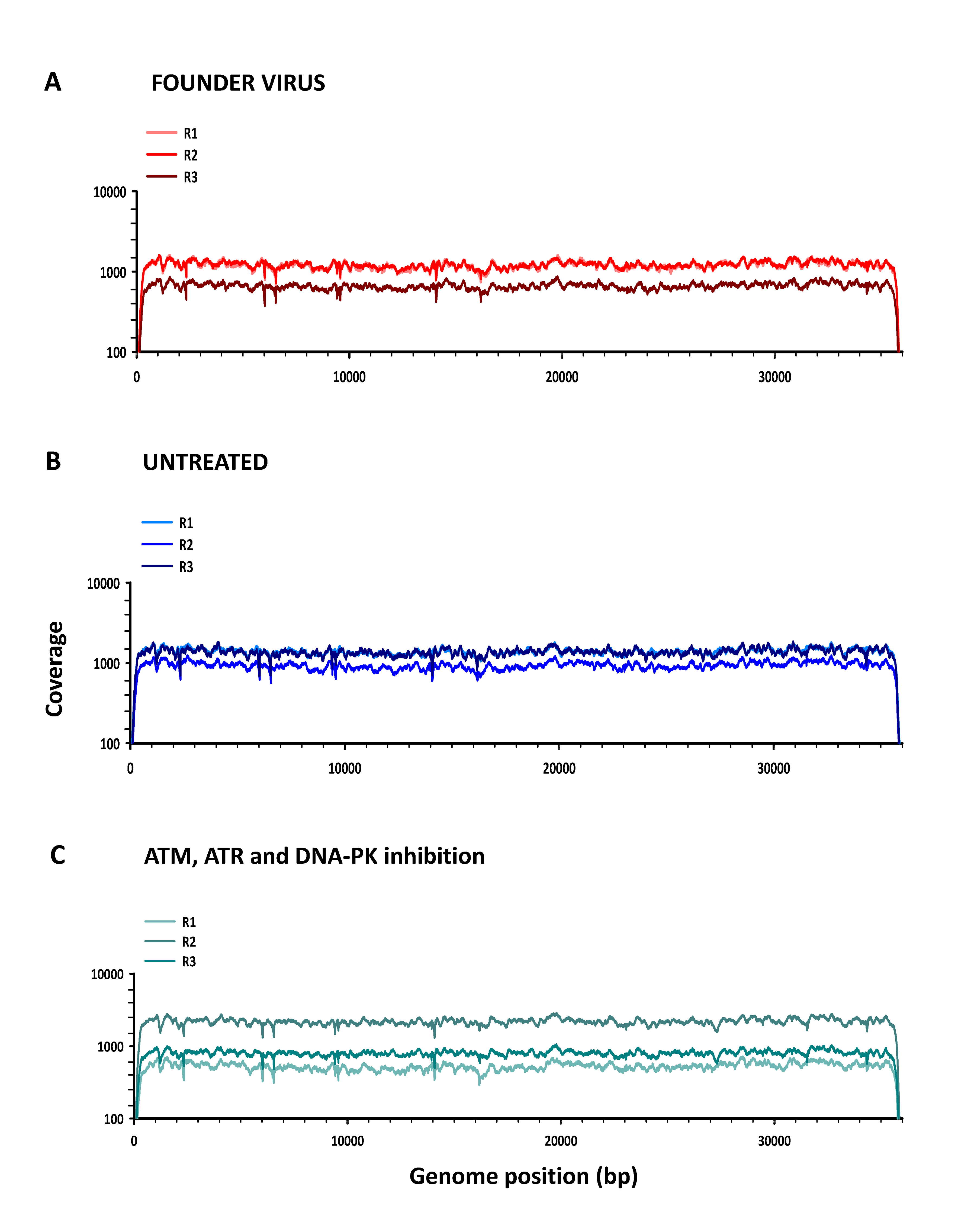

Supplement: Supplementary file 1 [file viruses-11-00938-s001.zip › Fig_S3.tiff]

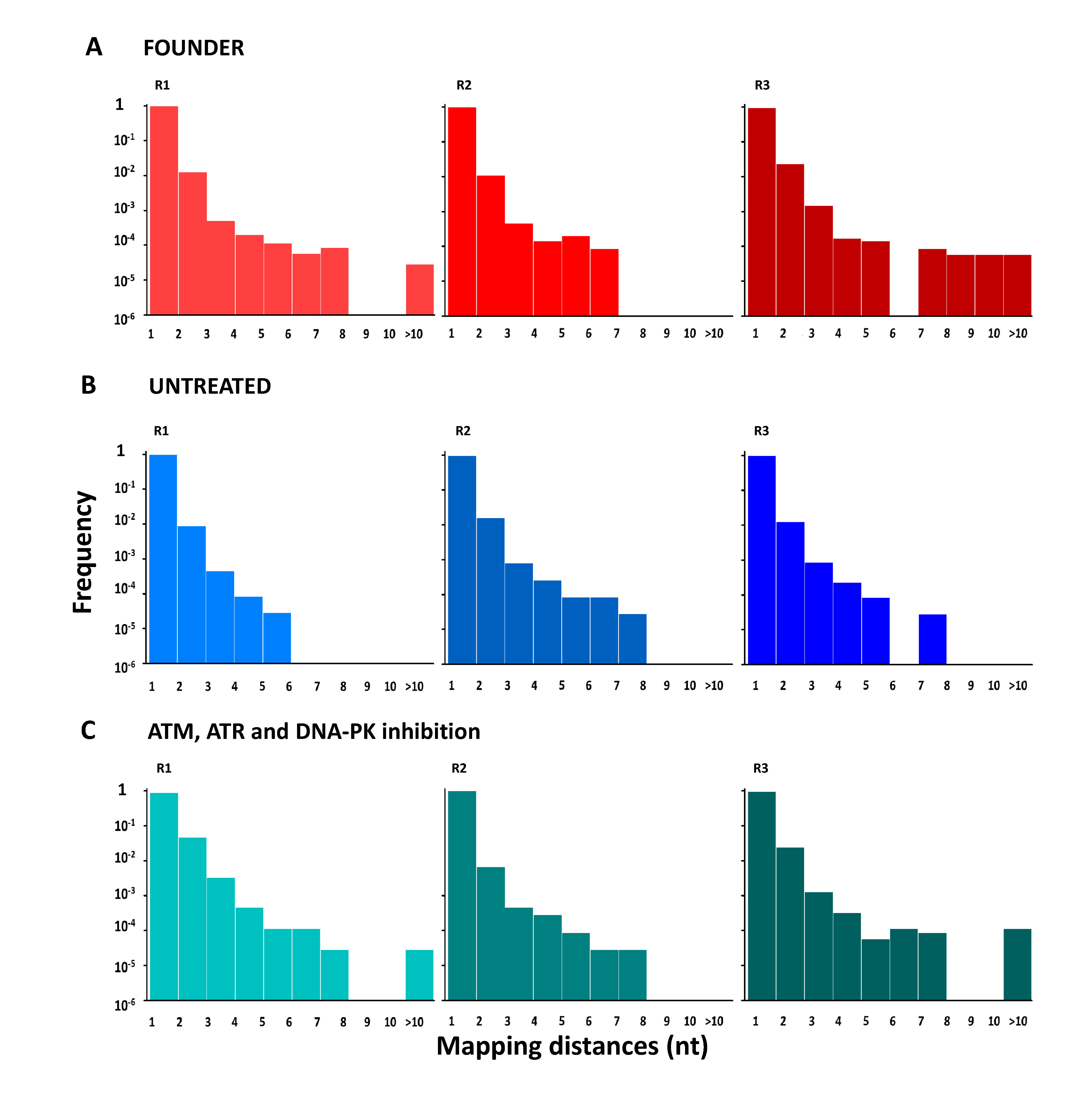

Supplement: Supplementary file 1 [file viruses-11-00938-s001.zip › Fig_S4.tiff]
